# Bipolar Disorder and Epilepsy: Clinical and Neurobiological Overlaps, Evidence Gaps, and an Exploratory Systems Perspective

**DOI:** 10.62641/aep.2406

**Published:** 2026-10-15

**Authors:** Rocío Gómez-Juanes, Mauro García-Toro, Joan Salvà

**Affiliations:** ^1^Research Network on Chronicity, Primary Care and Health Promotion (RICAPPS), Carlos III Health Institute, 28019 Madrid, Spain; ^2^University Institute of Health Science Research (IUNICS), University of the Balearic Islands, 07122 Palma, Spain; ^3^Health Research Institute of the Balearic Islands (IdISBa), 07120 Palma, Spain; ^4^Department of Medicine, University of the Balearic Islands, 07122 Palma, Spain; ^5^Department of Psychiatry, Son Espases University Hospital, 07120 Palma, Spain

**Keywords:** bipolar disorder, epilepsy, brain networks, neuroplasticity, neuroinflammation

## Abstract

**Background:**

Accumulating evidence suggests that bipolar disorder (BD) and epilepsy share important clinical, neurobiological, and therapeutic features. Both disorders are characterized by recurrent episodes and fluctuating clinical courses, raising the possibility that partially overlapping or analogous regulatory processes may contribute to some aspects of their clinical expression and progression. This review examines current evidence regarding the overlap between BD and epilepsy and explores integrative perspectives that may help explain their similarities.

**Methods:**

A narrative review of the literature was conducted. Evidence related to genetics, neuroimaging, cognition, neuroplasticity, neuroinflammation, stress biology, network neuroscience, and treatment response was examined.

**Results:**

Findings across BD and epilepsy reveal partially overlapping biological and clinical features, including alterations in excitation–inhibition balance, neuromodulatory disturbances, neuroinflammatory processes, and neuroplastic changes. Neuroimaging studies in both conditions reveal abnormalities in large-scale brain networks involved in emotional regulation, cognitive control, and behavioral adaptation. Clinically, both disorders show sensitivity to environmental and physiological stressors and sleep disruption, cognitive impairment, and partial responsiveness to overlapping pharmacological and neuromodulatory interventions. These observations are compatible with the hypothesis that recurrent episodes and prolonged network dysregulation may reinforce maladaptive patterns of instability through interacting feedback processes, although this interpretation remains to be tested directly.

**Conclusions:**

Current evidence identifies several clinical and biological similarities between BD and epilepsy but does not yet establish that these similarities reflect identical or directly shared causal mechanisms. An integrative perspective combining network dysfunction, neuroplasticity, and systemic regulatory processes may provide a useful heuristic framework for investigating disease heterogeneity, progression, and treatment response. Further longitudinal and comparative studies are required before this perspective can support personalized monitoring strategies or mechanism-informed interventions.

## Introduction

The association between psychiatric disorders and epilepsy has long been recognized in clinical practice [1, 2, 3, 4]. Population-based studies consistently show that individuals with epilepsy have substantially higher rates of psychiatric comorbidity than the general population, including depressive, anxiety, psychotic, and bipolar-spectrum disorders [5, 6]. Conversely, increased rates of seizure-related phenomena and neurological abnormalities have been reported among patients with bipolar disorder (BD) [7, 8]. Although psychosocial burden, treatment effects, and diagnostic difficulties may partly explain this overlap, shared biological vulnerabilities are also likely to contribute [9].

Advances in genetics, neuroimaging, and network neuroscience have transformed the understanding of complex brain disorders [10, 11]. Contemporary models increasingly emphasize large-scale neural networks and their capacity to maintain stable activity across changing internal and external conditions [12, 13]. From this perspective, recurrent seizures and mood episodes may reflect disturbances in distributed systems regulating excitability, emotional processing, cognition, and adaptive behavior. Several observations suggest partially overlapping clinical and biological features in BD and epilepsy, although it remains uncertain whether these similarities reflect shared mechanisms, analogous processes, or distinct pathways producing comparable clinical phenomena. Both disorders follow episodic courses in which relative stability is interrupted by pathological state transitions. Stress, sleep disruption, substance use, and physiological changes may precipitate episodes in both conditions, suggesting heightened sensitivity to biological and environmental perturbations [8, 14]. Experimental and clinical studies have identified similar alterations in BD and epilepsy, including changes in excitation–inhibition balance, neuromodulatory function, inflammatory signaling, neuroplasticity, and large-scale network organization. However, most of this evidence is indirect and does not establish that these alterations reflect identical causal mechanisms in the two disorders. [15, 16].

Neither BD nor epilepsy is biologically uniform. In BD, the relevance of neurobiological, cognitive, circadian, and inflammatory alterations may vary by subtype, mood polarity, mixed or psychotic features, illness stage, and treatment exposure [8]. Epilepsy is similarly heterogeneous in etiology, seizure type, age at onset, lesion burden, and epileptogenic network organization [2]. The similarities discussed here should therefore be understood as cross-cutting dimensions of vulnerability and recurrence rather than universal features of either disorder.

Cognitive dysfunction is another area of convergence. Deficits in attention, executive functioning, memory, and emotional regulation occur in both BD and epilepsy and may persist beyond acute episodes [17, 18]. These impairments contribute to occupational and social disability and reduced quality of life, indicating that both disorders affect broader brain functions beyond seizures or mood symptoms.

The use of some pharmacological and neuromodulatory interventions in both disorders provides an additional area of clinical convergence, but does not by itself demonstrate shared pathophysiological substrates, because treatment indications, targets, and mechanisms may differ between BD and epilepsy [19].

Examining both disorders together may therefore clarify how biological and psychosocial factors interact across illness trajectories and help identify mechanisms that transcend traditional diagnostic boundaries. However, observed similarities should not automatically be interpreted as evidence of common causation. They may reflect contributing factors, consequences of recurrent illness, treatment effects, nonspecific markers of disease burden, or partially shared final pathways. The present review accordingly identifies plausible areas of convergence while avoiding causal claims beyond the available evidence.

Previous reviews have addressed psychiatric comorbidity in epilepsy, the clinical association between epilepsy and BD, and overlapping pharmacological treatments [1, 4, 7]. Most, however, have focused on epidemiology, diagnosis, psychiatric manifestations in epilepsy, or selected therapeutic issues. This review extends that literature by integrating evidence across genetic, neurophysiological, network, inflammatory, cognitive, behavioral, and treatment domains, while distinguishing direct BD-epilepsy evidence from indirect and theoretical evidence.

This narrative review examines clinical and neurobiological similarities between BD and epilepsy and evaluates whether these findings may reflect partially overlapping, analogous, or disorder-specific mechanisms, without implying that seizures and mood episodes are equivalent. It proposes an integrative and translational framework linking partially shared principles of network instability, compensatory regulation, and recurrent state transitions with candidate biomarkers, temporal signatures, and intervention-response predictions. The framework is intended to generate testable hypotheses, guide longitudinal multimodal research, and support more individualized monitoring and treatment.

## Methods

Searches were conducted in PubMed/MEDLINE for publications from 1 January 2010 to 31 May 2026. Earlier seminal studies were included selectively when needed to contextualize foundational concepts such as kindling, sensitization, activity-dependent plasticity, and network instability. Search terms included combinations of “epilepsy,” “bipolar disorder,” “comorbidity,” “brain networks,” “network dysfunction,” “neuroplasticity,” “neuroinflammation,” “genetics,” “stress,” “neuromodulation,” “cognition,” and “treatment response.” Additional terms included “functional connectivity,” “connectomics,” “excitation-inhibition balance,” “GABA,” “glutamate,” “circadian rhythms,” “sleep,” “inflammation,” “mood stabilizers,” and “psychotherapy.” Boolean operators combined disorder-related terms with candidate mechanisms and therapeutic approaches. Additional publications were identified by screening the reference lists of selected articles and recent reviews.

Studies were eligible when they met the following criteria:

* Peer-reviewed, full-text publications written in English.

* Original studies, systematic reviews, meta-analyses, or clinically relevant narrative reviews examining BD, epilepsy, or both.

* Studies addressing at least one predefined domain: clinical overlap or comorbidity; genetic vulnerability; excitation-inhibition balance; neuromodulatory regulation; neuroplasticity; neuroinflammation; stress biology; large-scale brain network dysfunction; cognitive or emotional functioning; illness progression; or treatment response.

Studies were excluded when they met one or more of the following criteria:

* Conference abstracts, commentaries, protocols, theses, book chapters, case reports, or other non-peer-reviewed material.

* Studies focused exclusively on psychiatric or neurological conditions unrelated to BD, epilepsy.

* Studies lacking data or substantive discussion relevant to the predefined domains.

Given the breadth and heterogeneity of the literature, the review was not intended as a formal systematic synthesis. Its purpose was to integrate findings across levels of analysis into an exploratory framework for interpreting the clinical similarities between BD and epilepsy and identifying candidate processes potentially related to illness progression and treatment response.

Titles and abstracts were screened for relevance, after which potentially eligible full-text articles were assessed for methodological quality, relevance to the predefined domains, and contribution to the integrative aims of the review. Final inclusion was determined by author consensus. This process was designed to preserve the breadth required for a narrative review while ensuring a structured and critical synthesis of evidence from genetics, neuroimaging, neurophysiology, cognition, clinical epidemiology, and treatment research.

Particular attention was given to areas in which different methodological approaches converged on similar conclusions.

To limit overinterpretation, evidence was considered at three levels. Direct evidence comprised studies specifically examining comorbidity, bidirectional associations, genetic overlap, or shared clinical features between BD and epilepsy. Indirect evidence referred to findings obtained separately in each disorder that were considered potentially comparable at a descriptive level, including findings related to network connectivity, excitation-inhibition regulation, neuroinflammation, sleep disruption, or stress sensitivity. Theoretical hypotheses were used only to organize and interpret convergent observations and were not treated as direct empirical evidence of shared pathophysiology.

## Neurobiological Similarities and Potentially Overlapping Processes

### Genetic Vulnerability and Pleiotropy

Advances in genomic research have substantially improved our understanding of the biological relationship between BD and epilepsy. Although these conditions remain clinically distinct, growing evidence suggests that they share part of their genetic architecture [9, 20]. Large-scale genome-wide association studies and cross-disorder analyses have identified overlapping risk loci and biological pathways associated with epilepsy and several psychiatric disorders, including BD [9, 21]. These findings support the concept of pleiotropy, whereby individual genetic variants contribute to vulnerability across multiple clinical phenotypes rather than determining a single disorder.

Several pathways implicated in both conditions are involved in synaptic transmission, neuronal excitability, calcium signaling, neurodevelopment, and activity-dependent plasticity [9, 21]. Variants affecting ion channels, neurotransmitter systems, and intracellular signaling cascades may alter the way neural systems process, integrate, and regulate information, thereby increasing susceptibility to both seizures and affective instability.

Importantly, shared genetic influences should be viewed as sources of vulnerability rather than direct causes of specific symptoms. Individuals carrying similar risk variants may develop epilepsy, BD, both conditions, or neither, depending on the interaction between inherited susceptibility and environmental, developmental, and biological influences acting across the lifespan [9, 20, 22]. This perspective helps explain the considerable heterogeneity observed within both disorders and highlights the importance of examining mechanisms beyond the genetic level.

Although genetic findings cannot fully account for the clinical similarities between BD and epilepsy, they provide a biological basis for investigating whether partially overlapping or distinct processes operate at the level of neural circuits, brain networks, and systemic regulation.

### Excitation–Inhibition Balance and Neuromodulatory Dysfunction

The maintenance of a dynamic balance between neuronal excitation and inhibition is essential for normal brain function. Disturbances in this balance have long been recognized as central to epilepsy and are increasingly considered relevant to BD and other psychiatric disorders [23, 24, 25].

In epilepsy, excessive synchronization of neuronal activity is often associated with impaired inhibitory regulation within neural circuits. Contemporary models emphasize that this dysfunction is not simply the consequence of excessive excitation, but may involve abnormalities affecting inhibitory interneurons, receptor function, synaptic transmission, and local circuit organization [24, 25]. These alterations reduce the capacity of neural systems to maintain stable activity patterns and increase vulnerability to seizure generation.

Evidence from BD points to more subtle but potentially widespread disturbances in excitatory and inhibitory signaling. Neurochemical, pharmacological, and neuroimaging studies suggest alterations in glutamatergic and GABAergic neurotransmission, together with abnormalities affecting neuromodulatory systems such as dopamine and serotonin [16]. These neurotransmitter systems play critical roles in emotional regulation, reward processing, motivation, learning, and cognitive flexibility. Their dysregulation may therefore contribute to the emergence of depressive and manic states by altering the responsiveness of neural systems to internal and external stimuli [26, 27].

Importantly, inhibitory and neuromodulatory mechanisms are closely interconnected. Monoaminergic systems influence cortical excitability and synaptic plasticity, while inhibitory circuits help regulate the propagation and integration of neural signals [28, 29]. Rather than representing independent mechanisms, they form part of a broader regulatory architecture responsible for maintaining neural stability and adaptive flexibility. Although the relative contribution of these processes may differ between BD and epilepsy, disturbances in excitability and neuromodulatory regulation represent an important area of comparison.

Despite this partial overlap, the relative weight, spatial organization, and temporal expression of these regulatory disturbances differ substantially between the two disorders. In epilepsy, pathological activity is more directly linked to excessive local and regional synchronization, impaired inhibitory restraint, and rapid propagation through seizure-generating networks [24]. In BD, by contrast, abnormalities appear to involve more distributed and state-dependent dysregulation of fronto-limbic, salience, executive-control, and default-mode networks, together with disturbances in neuromodulatory gain, circadian regulation, emotional processing, and behavioral adaptation [27]. Thus, apparently similar alterations in excitation–inhibition balance or neuromodulatory signaling should not be interpreted as implying identical circuit dysfunction.

### Neuroplasticity, Neuroinflammation and Systemic Modulators

Beyond genetic vulnerability and neurotransmitter abnormalities, increasing evidence suggests that neuroplasticity, inflammatory processes, and systemic regulatory mechanisms play important roles in both BD and epilepsy [15, 30, 31].

Neuroplasticity allows the brain to adapt to changing environmental demands and life experiences. However, repeated exposure to pathological states may also promote maladaptive changes that increase future vulnerability. In epilepsy, recurrent seizures have been associated with structural and functional reorganization of neural circuits that may facilitate subsequent seizure activity [31]. Similarly, repeated mood episodes in BD have been linked to persistent alterations in neural systems involved in emotional regulation and cognitive control [32, 33, 34]. These observations are consistent with concepts such as sensitization, kindling, and neuroprogression, which propose that recurrent episodes may increase susceptibility to future recurrences [35, 36].

Inflammatory mechanisms represent another potentially relevant area of convergence. In epilepsy, neuroinflammatory processes have been implicated in epileptogenesis and seizure susceptibility through effects on neuronal excitability, glial activation, and blood–brain barrier integrity [15]. In BD, low-grade systemic inflammation has been repeatedly associated with acute mood episodes and illness severity and may influence neurotransmission, neuroplasticity, and cellular metabolism [8]. However, the direction and causal significance of these associations remain uncertain, and inflammatory alterations may vary according to illness stage, medication exposure, metabolic status, sleep disruption, and comorbid medical conditions [8, 15].

Several systemic factors may further modulate these processes. Dysregulation of the hypothalamic–pituitary–adrenal axis, sleep and circadian disturbances, metabolic abnormalities, and gut–brain interactions have all been implicated in epilepsy and affective disorders [8, 14]. Gut–brain interactions may also modulate inflammatory, metabolic, and neuroendocrine processes relevant to bipolar disorder and epilepsy, although the available evidence remains heterogeneous and is stronger for disorder-specific associations than for directly shared mechanisms [37, 38, 39]. Chronic stress, for example, may alter neuroendocrine activity, inflammatory responses, and synaptic plasticity, thereby increasing susceptibility to seizures or mood episodes. Likewise, sleep disruption and circadian misalignment are well-established precipitants of both seizure recurrence and mood destabilization [2, 32].

Taken together, these findings suggest that neuroplastic, inflammatory, and neurohumoral alterations may interact with vulnerability and recurrence in both disorders. However, the current evidence does not establish that these processes operate through identical pathways, nor does it determine whether they are primary drivers, downstream consequences, or nonspecific correlates of recurrent illness. It is worth noting that this integrated view provides a conceptual basis to the testable predictions outlined below: if reciprocal amplification among plastic, inflammatory, and neurohumoral processes contributes to episodic instability, then longitudinal changes in neuroplastic markers, inflammatory signaling, and endocrine–immune regulation should precede clinical transitions and represent candidate targets for mechanism-informed interventions [38, 40, 41] (see Fig. 1).

**Fig. 1.**
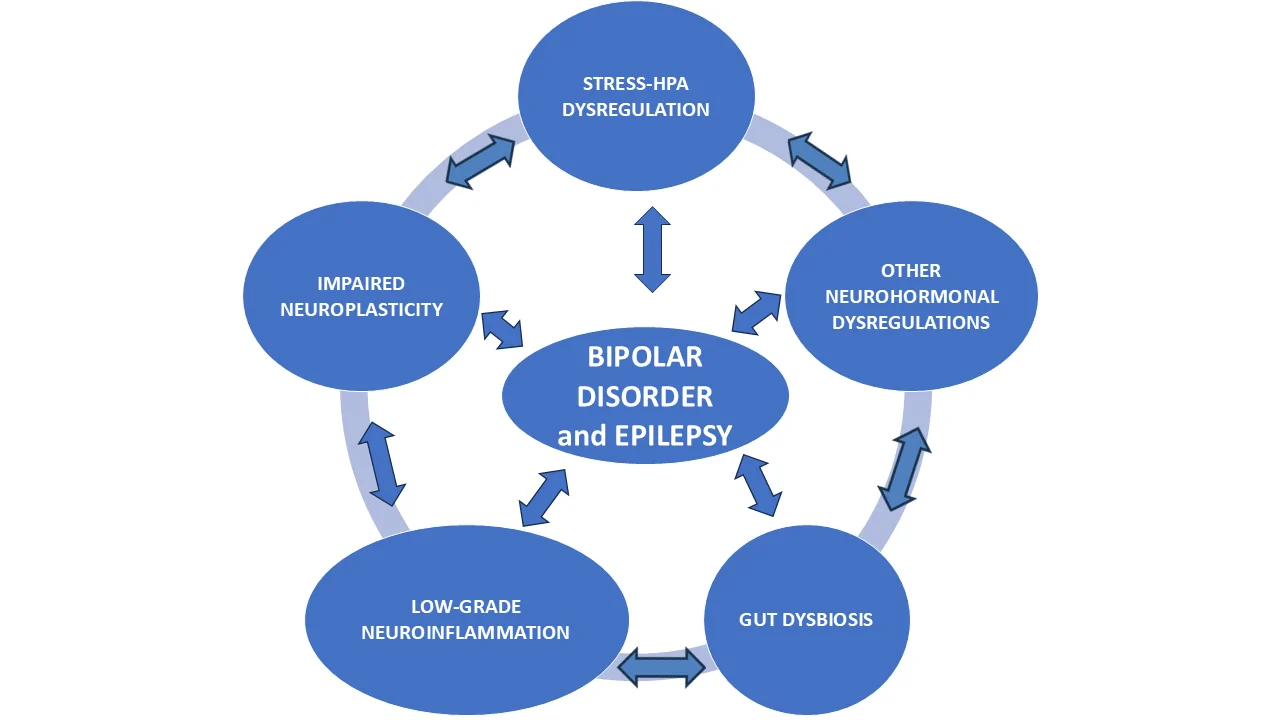
**Hypothesized interactions among systemic modulators potentially relevant to bipolar disorder and epilepsy**. These processes represent candidate domains for comparative and translational research, although their shared causal and therapeutic significance remains to be established.

Considered together, current evidence identifies partially overlapping biological findings in BD and epilepsy across levels ranging from genetic susceptibility to systemic regulation. While these mechanisms operate at different levels of organization, their effects are ultimately expressed through the activity of distributed neural systems. Consequently, examining how large-scale brain networks maintain—or fail to maintain—stable patterns of function may provide important insights into the recurrent and heterogeneous nature of both disorders.

## Brain Network Dysfunction and State Transitions

### Network Instability as an Organizing Perspective

Network neuroscience indicates that both bipolar disorder and epilepsy are associated with alterations in the organization and regulation of large-scale brain networks [13, 31]. These findings are compatible with network-based models but do not establish network abnormalities as primary causal mechanisms in either disorder.

The specific alterations likely differ across clinical subtypes. In epilepsy, network organization depends on etiology, seizure type, epileptogenic focus, and the extent of propagation beyond the primary seizure-generating region [42]. In BD, network abnormalities may vary with bipolar subtype, current mood state, psychotic features, age at onset, episode burden, and medication status [43]. Network instability should therefore be understood as a broad organizing principle rather than a single anatomical or functional signature shared by all forms of both disorders.

The network perspective has been particularly influential in epilepsy. Seizures are increasingly viewed as events arising from interactions among cortical and subcortical regions rather than solely from localized foci [24, 44]. Structural and functional connectivity studies indicate that seizure generation, propagation, and termination depend on the dynamic organization of widespread neural circuits [12, 44, 45]. A similar shift has occurred in BD research. Neuroimaging studies reveal abnormalities in networks involved in emotional regulation, salience attribution, reward processing, and executive control [13]. Altered prefrontal-limbic connectivity has been associated with mood instability, impaired emotional regulation, and cognitive dysfunction, supporting distributed rather than region-specific models of the disorder.

In both conditions, network abnormalities appear dynamic rather than fixed. Connectivity patterns may fluctuate across illness phases and may partially normalize after effective treatment or sustained remission [31, 33]. This variability is particularly relevant to disorders characterized by recurrent episodes and heterogeneous clinical trajectories.

### State Transitions and Threshold Effects

One of the clearest similarities between BD and epilepsy is their episodic course. Both involve periods of relative stability interrupted by transitions to pathological states, including seizures in epilepsy and depressive, manic, or hypomanic episodes in BD [2, 32].

In epilepsy, experimental and clinical studies suggest that seizures may be preceded by progressive changes in neural activity before a critical threshold is reached [46]. However, the reliability and clinical utility of these preictal signatures vary across epilepsy syndromes and recording methods. In BD, some mood episodes are preceded by prodromal changes in sleep, energy, activity, emotional reactivity, or cognition. Such changes may signal an approaching transition in a subgroup of patients, but they do not establish a uniform biological sequence or causal pathway [47]. Nevertheless, recognizable prodromal phases suggest that regulatory disturbances may accumulate before symptoms become fully apparent.

Sleep deprivation, psychosocial stress, circadian disruption, substance use, and medical illness may modulate transition risk in both disorders [14, 15]. Their clinical relevance is discussed further in next section.

The tendency for recurrent episodes to become more frequent or more easily triggered in some individuals suggests that vulnerability may change over time [35, 36, 48, 49]. Characterizing these transitions may therefore help identify early warning signs and support preventive interventions.

### Neuroimaging Evidence for Network Abnormalities in BD and Epilepsy

Neuroimaging provides important support for network-based models of BD and epilepsy. Structural and functional studies have identified abnormalities in connectivity, network efficiency, and communication among distributed brain regions in both disorders [33, 44].

In epilepsy, alterations commonly involve temporo-limbic and thalamo-cortical circuits related to seizure generation and propagation, although affected networks vary by epilepsy subtype and epileptogenic focus [31]. Many abnormalities extend beyond the presumed focus, emphasizing the distributed nature of epileptic processes. In BD, neuroimaging consistently implicates fronto-limbic systems involved in emotional regulation, together with salience, executive-control, and default-mode networks [13, 34]. Altered communication between prefrontal regions and limbic structures, particularly the amygdala, is among the most replicated findings and has been associated with impaired top-down regulation, affective instability, and recurrent mood episodes [50, 51].

Despite circuit-specific differences, studies in both disorders have reported altered connectivity, reduced flexibility, and impaired integration across distributed systems. Vulnerability may therefore arise not only from abnormalities within individual regions but also from disrupted communication among functional networks.

Longitudinal studies suggest that network organization may change over the course of illness. In epilepsy, seizure burden and illness duration have been associated with progressive connectivity alterations, some of which may improve after successful treatment [31]. In BD, repeated mood episodes have likewise been linked to structural and functional connectivity changes, although findings remain heterogeneous [33, 52, 53].

Overall, both disorders involve large-scale network disturbances. Although the affected systems vary by phenotype, altered connectivity, reduced adaptability, and vulnerability to pathological transitions represent useful dimensions for comparison. These abnormalities may also contribute to the cognitive, emotional, and behavioral difficulties considered in the following section.

### Divergent Circuit and Oscillatory Dynamics

Although BD and epilepsy may share broad principles of network instability, their pathological transitions differ markedly in circuit organization and electrophysiological dynamics [46, 54, 55]. Epileptic seizures typically involve abrupt transitions toward hypersynchronous activity within seizure-generating networks. Depending on epilepsy type and localization, temporo-limbic, thalamo-cortical, frontal, or other cortical-subcortical circuits may be involved, followed by recruitment of wider propagation networks. High-frequency activity, interictal epileptiform discharges, phase-synchronization changes, and nonlinear dynamic shifts have been investigated as markers of epileptogenic tissue and seizure onset [55].

Mood episodes in BD evolve over longer time scales and are not defined by a discrete hypersynchronous event. Evidence instead suggests altered coordination among fronto-limbic, salience, executive-control, and default-mode networks, accompanied by disturbances in reward, cognition, emotional regulation, sleep, and circadian rhythms [27]. Electroencephalographic (EEG) studies report oscillatory and connectivity abnormalities, but findings vary across mood states, medication exposure, illness stage, and analytic methods [56]. These should therefore be considered candidate correlates of large-scale network dysregulation rather than analogues of ictal activity.

This distinction is clinically important. Seizures can be conceptualized as relatively rapid, spatially organized transitions into pathological synchronization, whereas mood episodes involve more prolonged disturbances in distributed affective, cognitive, behavioral, and circadian regulation. The framework therefore uses vulnerability, compensation, and recurrence as comparative dimensions rather than implying equivalence between seizures and mood episodes.

Despite their different spatial and temporal dynamics, these disturbances may contribute to overlapping impairments in cognition, emotional regulation, behavioral adaptation, and long-term functioning [57, 58]. These dimensions are examined in the following section.

## Clinical and Cognitive Overlap

### Cognitive Dysfunction Beyond Acute Episodes

Cognitive impairment is a major contributor to disability in both BD and epilepsy. Although traditionally viewed as a secondary consequence of seizures or mood episodes, persistent deficits are frequently observed during periods of relative clinical stability [18, 59].

In epilepsy, cognitive dysfunction commonly affects attention, executive functioning, processing speed, and memory. Its severity may depend on seizure burden, age at onset, etiology, and treatment exposure. However, deficits may persist despite satisfactory seizure control, suggesting broader alterations in brain function beyond the immediate effects of seizures [18]. Similarly, many individuals with BD show persistent deficits in executive functioning, verbal memory, attention, and cognitive flexibility despite remission of mood symptoms [17, 59]. These difficulties may impair occupational performance, interpersonal relationships, and treatment adherence, contributing substantially to long-term disability.

In both disorders, cognitive dysfunction appears to reflect stable vulnerabilities combined with state-dependent fluctuations. Cognitive performance may worsen during acute illness phases and may remain impaired afterward in some patients. However, it remains uncertain whether persistent deficits reflect cumulative effects of recurrent episodes, pre-existing vulnerability, treatment exposure, illness severity, or other associated factors [60, 61].

### Emotional Regulation and Behavioral Vulnerability

Both BD and epilepsy are also associated with difficulties in emotional regulation and behavioral adaptation. In BD, emotional dysregulation contributes to mood instability, impulsivity, interpersonal difficulties, and relapse risk [62, 63]. Increased emotional reactivity and difficulty managing negative affect may persist during euthymia.

Individuals with epilepsy also have elevated rates of depression, anxiety, irritability, and emotional instability [64]. These symptoms may occur independently of seizure frequency and contribute substantially to illness burden and reduced quality of life. In some patients, emotional disturbances precede seizure worsening, while recurrent seizures further increase psychological distress, suggesting a bidirectional relationship [65].

The coexistence of cognitive and emotional dysfunction highlights the interaction between executive-control and emotion-processing systems. Disturbances in these interconnected networks may therefore contribute to clinical manifestations extending beyond the defining features of either disorder [13, 31, 50, 63, 64, 65].

### Stress as a Modifier of Illness Course

Stress is among the most consistently identified factors associated with episode occurrence, recurrence, and symptom severity in both BD and epilepsy. Numerous studies have demonstrated associations between psychosocial stress and increased risk of seizures, mood episodes, symptom worsening, and functional decline [14, 32, 65].

Of note, stress may operate both as an acute precipitant and as a clinically relevant modifier of vulnerability. Its effects are likely to interact with sleep quality, emotional regulation, cognitive functioning, treatment adherence, and social circumstances [66, 67]. Although stress-related neuroendocrine and inflammatory mechanisms were discussed previously, from a clinical perspective the main implication is that sustained stress exposure may contribute to patterns of symptom worsening, reduced functioning, and increased recurrence risk that could become self-reinforcing over time.

### Sleep, Circadian Rhythms, Lifestyle, and Modifiable Clinical Factors

Sleep, circadian regularity, physical activity, dietary habits, substance use, and social support are clinically relevant because they may influence the expression of pre-existing vulnerability rather than constitute primary causes of either disorder.

Sleep disturbances represent one of the clearest areas of clinical convergence between BD and epilepsy. Sleep deprivation is a recognized precipitant of seizures and mood episodes, while circadian disruption has been particularly implicated in mood instability and may also influence seizure susceptibility [14, 58]. In BD, disturbances of sleep and circadian rhythms are considered central components of the illness and often precede mood episodes. Similarly, inadequate sleep is associated with lower seizure thresholds and increased seizure frequency in epilepsy. These observations support the clinical importance of maintaining regular sleep and circadian patterns [68].

Lifestyle factors may also influence disease trajectories. Physical inactivity, unhealthy dietary patterns, substance misuse, and limited social support have each been associated with poorer outcomes in one or both disorders [69, 70]. Conversely, interventions promoting healthy lifestyle habits, including regular physical activity, sleep stabilization, stress management, and dietary improvement, may improve psychological well-being and functional outcomes, although evidence for direct effects on seizure frequency or mood-episode recurrence remains variable and disorder-specific [69, 70]. Although lifestyle factors are unlikely to represent primary causes of either disorder, they may substantially influence the way biological vulnerabilities are expressed in everyday life. This makes them particularly relevant targets for preventive and adjunctive interventions.

### Functional Outcomes and Illness Progression

Despite considerable heterogeneity, both BD and epilepsy are associated with significant long-term functional consequences. Recurrent episodes may interfere with education, employment, interpersonal relationships, and overall quality of life, often resulting in substantial social and economic burden [8, 18, 32].

Illness course may be associated not only with the frequency of seizures or mood episodes but also with cognitive difficulties, emotional dysregulation, stress exposure, and impaired adaptive functioning. However, the direction and causal relationships among these factors remain uncertain. Earlier age at onset, greater episode burden, and persistent cognitive difficulties have been associated with less favorable outcomes in studies of BD and epilepsy, although the strength and consistency of these associations differ between disorders [31, 33].

At the same time, considerable variability exists among patients. Some individuals experience long periods of stability and maintain high levels of functioning, whereas others develop increasingly recurrent and disabling illness trajectories. Understanding the factors that contribute to these divergent outcomes remains an important priority for future research.

Viewed collectively, the evidence reviewed in this section identifies cognitive, emotional, and behavioral difficulties in both BD and epilepsy that extend beyond their defining symptoms, while their underlying mechanisms and clinical expression may differ (see Table 1).

**Table 1.** **Clinical and neurobiological similarities between bipolar disorder and epilepsy**.

Domain	Epilepsy	Bipolar disorder	Potentially comparable features
Clinical course	Recurrent seizures separated by interictal periods	Recurrent depressive, manic, or hypomanic episodes	Episodic course with periods of stability and recurrence
Genetic factors	Polygenic susceptibility involving neuronal excitability and synaptic function	Polygenic susceptibility involving neurotransmission and neurodevelopment	Partial genetic overlap and pleiotropic risk factors
Neural excitability	Abnormal synchronization and reduced inhibitory control	Altered excitatory–inhibitory balance and neuromodulatory dysfunction	Alterations in processes involved in neural stability
Neuroplasticity	Circuit reorganization associated with recurrent seizures	Neuroprogressive changes associated with recurrent mood episodes	Activity-dependent plasticity influencing future vulnerability
Inflammation	Inflammation contributes to epileptogenesis and seizure susceptibility	Low-grade inflammation associated with episodes and illness severity	Inflammation may amplify underlying vulnerabilities
Brain networks	Altered connectivity within seizure-related networks	Altered connectivity within fronto-limbic and regulatory networks	Disturbances affecting large-scale neural systems
Cognition	Deficits in attention, memory, and executive function	Deficits in attention, memory, and executive function	Persistent cognitive impairment beyond acute episodes
Stress sensitivity	Stress may precipitate seizures	Stress may precipitate mood episodes	Increased vulnerability to stressors
Sleep and circadian rhythms	Sleep disruption lowers seizure threshold	Sleep disruption frequently precedes mood episodes	Circadian instability associated with symptom recurrence
Treatment response	Antiseizure drugs, neuromodulation, psychosocial and lifestyle interventions	Mood stabilizers, neuromodulation, psychosocial and lifestyle interventions	Partial overlap in therapeutic approaches and candidate targets

## Therapeutic Overlap and Potential Mechanistic Implications

The therapeutic overlap between BD and epilepsy represents a clinically relevant area of comparison. Several interventions are used in both disorders, although their indications may differ. Their effects on neural excitability, neuroplasticity, network regulation, or stress-related processes provide hypotheses for comparative research [8, 19]. However, therapeutic overlap should not be interpreted as evidence that the two disorders share identical treatment mechanisms or that efficacy can be generalized directly from one condition to the other. The indications, level of evidence, optimal treatment targets, safety considerations, and expected clinical outcomes differ substantially between BD and epilepsy.

### Pharmacological Overlap

A clinically important example of therapeutic overlap is the use of selected antiseizure medications in the treatment of BD. Valproate, carbamazepine, and lamotrigine are prescribed in both BD and epilepsy and have demonstrated efficacy for specific indications within each disorder [2, 70]. Nevertheless, this overlap is partial and clinically specific. In epilepsy, medication selection depends primarily on seizure type, epilepsy syndrome, etiology, comorbidities, and the risk of seizure aggravation. In BD, the effectiveness of these agents differs according to episode polarity, illness phase, relapse-prevention objectives, and individual clinical characteristics. For example, lamotrigine has a more established role in the prevention of depressive episodes than in acute mania, whereas valproate and carbamazepine are more frequently used in the management of manic states or maintenance treatment [71, 72, 73].

Despite differences in their pharmacological profiles, these medications influence several processes implicated in both disorders, including neuronal excitability, synaptic transmission, intracellular signaling, and activity-dependent plasticity. Their effectiveness in different diagnostic contexts raises questions about potentially comparable regulatory processes. This interpretation should remain cautious, as common pharmacological effects do not establish a common causal mechanism. This distinction is illustrated by the fact that some antiseizure medications have established efficacy in epilepsy but have little or no role in BD, whereas lithium and several atypical antipsychotics are central treatments for BD but are not antiseizure therapies [74, 75]. Even among shared agents, clinical utility differs substantially: lamotrigine has an established role in maintenance treatment and prevention of depressive recurrence in BD, whereas in epilepsy it is prescribed according to seizure phenotype [71, 75].

At the same time, therapeutic response is highly heterogeneous. Not all patients benefit from the same interventions, and considerable variability exists in efficacy, tolerability, and long-term outcomes. This heterogeneity underscores the need for more individualized approaches that consider the diversity of biological and clinical profiles observed within both disorders.

### Neuromodulation and Circuit-Based Interventions

Neuromodulation is increasingly used in both neurology and psychiatry. Vagus nerve stimulation (VNS), transcranial magnetic stimulation (TMS), deep brain stimulation (DBS), and electroconvulsive therapy (ECT) have demonstrated efficacy in selected patients with epilepsy, mood disorders, or both [76, 77, 78, 79]. However, evidence and indications differ. VNS and DBS have established roles in selected forms of drug-resistant epilepsy, whereas TMS and ECT have stronger evidence in mood disorders, including bipolar depression and severe treatment-resistant episodes. Their targets, stimulation parameters, and expected outcomes also differ [80, 81, 82].

These interventions directly modulate neural circuits and may provide information about how changes in network activity relate to clinical outcomes. Their effects appear to involve not only local stimulation but also broader changes in connectivity, synchronization, and communication across distributed systems [50, 83]. Nevertheless, their ability to influence networks in both disorders does not prove shared disease-specific circuitry. Effects vary according to anatomical target, underlying pathology, stimulation protocol, and clinical objective, including seizure reduction, mood stabilization, or cognitive improvement [84].

The clinical effects of neuromodulation are consistent with the relevance of large-scale network activity to treatment response in epilepsy and mood disorders, but do not establish equivalent network pathology. These approaches illustrate how circuit-level interventions can affect complex clinical phenomena across neurological and psychiatric conditions. Treatment selection, however, should remain based on disorder-specific evidence, current recommendations, and individualized risk-benefit assessment.

### Psychosocial Interventions and Stress Reduction

Psychosocial interventions also play an important role in the management of both BD and epilepsy. In BD, psychoeducation, cognitive-behavioral therapy, family-focused therapy, and interpersonal and social rhythm therapy have demonstrated benefits in reducing relapse risk, improving treatment adherence, and enhancing psychosocial functioning [85, 86, 87]. Although these interventions differ in their specific objectives, many share a common focus on reducing stress, improving self-regulation, and stabilizing daily routines.

Similarly, psychosocial interventions in epilepsy have been associated with improvements in quality of life, emotional well-being, and, in some studies, reductions in seizure frequency [88, 89]. These benefits are likely multifactorial but may partly reflect reduced exposure to stress-related physiological and behavioral triggers. The evidence base is more robust for structured psychosocial interventions in BD than for seizure-related outcomes in epilepsy. Therefore, psychosocial approaches in epilepsy should primarily be considered adjunctive strategies for improving coping, emotional well-being, adherence, quality of life, and management of seizure-related stress, rather than substitutes for antiseizure treatment.

Particular interest has recently focused on complementary use of mindfulness-based stress reduction interventions. Preliminary evidence suggests that mindfulness training may improve emotional regulation, cognitive functioning, and perceived stress in individuals with BD, although not all studies are consistent [90, 91]. Emerging studies in epilepsy have reported improvements in psychological well-being and possible beneficial effects on seizure-related outcomes, although larger controlled studies are still needed [92, 93]. Accordingly, mindfulness-based interventions should be interpreted as promising but still adjunctive approaches in both conditions. Current evidence does not support assuming equivalent efficacy for mood stabilization in BD and seizure control in epilepsy.

### Lifestyle Interventions and Biological Rhythm Stabilization

Lifestyle interventions have attracted increasing attention as adjunctive treatments for both BD and epilepsy. This interest reflects growing recognition that sleep quality, physical activity, diet, and social rhythms are associated with biological and psychosocial factors relevant to illness course.

Sleep stabilization is particularly relevant. Given the well-established role of sleep disruption in precipitating seizures and mood episodes, interventions aimed at improving sleep quality and maintaining regular circadian rhythms may support symptom management and general functioning [14, 94].

Physical activity has also been associated with improvements in mood, cognitive functioning, and overall quality of life in both neurological and psychiatric populations [95]. In addition, increasing attention has been directed toward nutritional factors and metabolic health, particularly considering growing evidence linking inflammation, mitochondrial energy metabolism, and neural function in mood disorders [96].

However, the strength and clinical relevance of the evidence differ between interventions and disorders. In BD, sleep and social-rhythm stabilization may contribute to relapse prevention and mood regulation, whereas in epilepsy the main aims may include reduction of modifiable seizure triggers, improvement of general health, and enhancement of quality of life. Evidence for direct effects of diet, exercise, or stress-management interventions on core seizure or mood outcomes remains more limited and heterogeneous than those supporting established pharmacological treatments [69, 95].

Although the evidence base remains more limited than for pharmacological treatments, lifestyle interventions may influence several behavioral, psychosocial, and potentially biological factors relevant to long-term management. As a result, they may represent valuable components of comprehensive long-term management strategies [97]. Their role should nevertheless be framed as complementary to, rather than interchangeable with, disorder-specific pharmacological care.

### Implications for Personalized and Multilevel Care

The therapeutic overlap observed between BD and epilepsy may have implications for comparative research and for the development of individualized, multilevel care. Rather than focusing exclusively on symptom suppression, effective interventions may influence several interacting clinical and biological processes, including neural excitability, neuroplasticity, stress responsiveness, emotional regulation, and biological rhythm stability, although their relative importance and causal role remain uncertain. Recent developments in BD and epilepsy increasingly support precision-oriented treatment models that integrate disorder-specific mechanisms with potentially modifiable clinical factors rather than focusing exclusively on isolated symptoms [32, 98, 99]. This perspective does not imply that the same intervention should be used in both disorders on the basis of putative shared mechanisms. Instead, it suggests that some transdiagnostic modifiers—such as sleep disruption, chronic stress, cognitive difficulties, poor adherence, and lifestyle instability—may be clinically relevant across both conditions and can be addressed alongside disorder-specific treatment. This perspective also supports the development of multilevel treatment strategies that combine pharmacological, psychological, lifestyle, and neuromodulatory interventions according to individual patient characteristics. Such approaches may be particularly valuable in patients with recurrent episodes, persistent functional impairment, or incomplete response to standard therapies [32].

Treatment decisions should remain individualized and based on current disorder-specific guidelines, illness phase, seizure type where applicable, mood polarity, psychiatric and neurological comorbidities, medication interactions, contraindications, reproductive considerations, patient preferences, and the availability of specialized care. A better understanding of treatment mechanisms may facilitate the development of more personalized, preventive, and mechanism-informed interventions capable of improving long-term outcomes in both disorders [98]. However, therapeutic overlap should be understood as a source of hypotheses and potential clinical complementarity, rather than as evidence of directly transferable efficacy or identical pathophysiology.

## An Exploratory Integrative Systems Hypothesis

The evidence reviewed in this article identifies several biological and clinical similarities between BD and epilepsy that may not be fully captured by isolated risk factors or single-mechanism accounts. Genetic vulnerability, altered neural excitability, neuroplastic changes, inflammation, stress-related processes, network dysfunction, and lifestyle factors have each been implicated in the expression or course of BD, epilepsy, or both [39, 57, 100, 101, 102, 103, 104]. A major challenge is to understand how these factors interact over time and how they may contribute to recurrent seizures or mood episodes in different clinical contexts.

The added value of this perspective is not to replace established disorder-specific models or to infer identical pathophysiology from clinical similarities. Rather, it is to provide a framework for integrating findings that are usually examined separately in epilepsy and BD research. Previous reviews have often emphasized psychiatric comorbidity and diagnostic or pharmacological overlap. By contrast, the present review combines direct, indirect, and theoretical evidence across multiple levels of analysis and relates this synthesis to candidate measures of network instability, biological regulation, behavioral change, and clinical transition. This approach may be particularly useful for generating longitudinal hypotheses concerning recurrence, heterogeneity, and treatment response.

Thus, a systems-level perspective may help organize these findings without replacing established disorder-specific models (see Table 2).

**Table 2.** **Candidate processes potentially relevant to clinical and biological similarities between bipolar disorder and epilepsy**.

Mechanism	Evidence in epilepsy	Evidence in bipolar disorder	Potential clinical relevance
Shared genetic vulnerability	Overlapping risk loci affecting neuronal signaling	Shared susceptibility genes identified in cross-disorder studies	May contribute to comorbidity and phenotypic heterogeneity
Excitation–inhibition imbalance	Central mechanism in seizure generation	Altered glutamatergic and GABAergic signaling	May influence recurrence and treatment response
Neuromodulatory dysfunction	Modulates seizure susceptibility and network stability	Dopaminergic and serotonergic dysregulation linked to mood states	May contribute to state transitions and symptom variability
Maladaptive neuroplasticity	Recurrent seizures promote circuit reorganization	Repeated mood episodes associated with neuroprogression	May increase vulnerability to future episodes
Neuroinflammation	Involved in epileptogenesis and seizure recurrence	Associated with mood episodes and illness severity	Potential domain for therapeutic research in both disorders
Stress-system dysregulation	HPA-axis alterations influence seizure susceptibility	HPA-axis abnormalities associated with mood instability	May mediate the effects of psychosocial stress
Sleep and circadian disruption	Facilitates seizure occurrence	Frequently precedes mood episodes	Important target for prevention strategies
Network dysfunction	Altered connectivity and synchronization	Altered fronto-limbic and regulatory network function	May be associated with episodic transitions and cognitive impairment
Reciprocal feedback processes [Schismogenesis]	Interactions among seizures, stress, and neuroplasticity	Interactions among mood episodes, stress, and neuroplasticity	Potential framework for understanding recurrence and progression

In epilepsy, epileptogenesis, excitation–inhibition imbalance, seizure-network organization, neuroinflammation, and structural or functional reorganization provide important mechanistic accounts of seizure susceptibility and recurrence. In BD, neuroprogression, sensitization, circadian dysregulation, allostatic load, and fronto-limbic network models offer complementary explanations for mood instability and illness progression.

Within this context, complementary schismogenesis is introduced as an exploratory higher-level hypothesis. It does not posit a distinct biological mechanism shared by BD and epilepsy. Rather, it suggests that, in some individuals, repeated interactions between pathological activation and compensatory inhibitory regulation may become increasingly differentiated and mutually reinforcing over time. This idea may be relevant when recurrent episodes are accompanied by persistent changes in stress responsiveness, sleep regulation, cognition, inflammatory activity, or network function.

The framework should therefore be understood as a heuristic for generating hypotheses about reciprocal feedback processes, not as an empirically validated account of disease pathogenesis. It does not imply that seizures and mood episodes are equivalent phenomena, that they arise from the same circuits, or that they follow identical biological trajectories. From this perspective, BD and epilepsy may be examined as disorders involving disturbances in network regulation. In both conditions, the nervous system may become less able to maintain a stable balance between activation and inhibition. The biological basis of this instability is likely to differ between disorders. In many forms of epilepsy, impaired regulation may involve disturbances in neuronal inhibition, including alterations in GABAergic mechanisms. In BD, candidate processes include alterations in neuromodulatory systems, including dopaminergic and serotonergic pathways [16, 105]. Within the proposed framework, sustained impairment of regulatory capacity may permit the emergence of self-reinforcing pathological dynamics. In epilepsy, this may involve local or regional failures of inhibitory restraint that facilitate hypersynchronization and seizure propagation. In BD, it may involve distributed alterations in neuromodulatory regulation, fronto-limbic coordination, circadian stability, and adaptive control. These proposals should be interpreted as theoretical possibilities rather than as established causal mechanisms. A more general possibility is that persistent failures of regulatory or compensatory processes may permit the emergence of relatively self-reinforcing patterns of pathological activity in both disorders, although the biological basis of these processes is likely to differ. If compensatory responses are insufficient or become persistent, they could hypothetically contribute to the stabilization of maladaptive patterns of regulation. Within this hypothetical state, some functional domains may become relatively overactive, whereas others may become relatively underactive or subject to excessive compensatory inhibition [106]. Repeated cycles of activation and compensation may progressively reinforce this imbalance. This proposed dynamic is consistent with the systemic concept of complementary schismogenesis, in which two opposing but interdependent processes become increasingly differentiated while continuing to influence each other [40].

In clinical terms, this framework may provide one way of interpreting the coexistence of acute episodes or seizures with persistent inter-episode or interictal alterations. Repeated seizures or mood episodes have been associated with changes in neuroplasticity, inflammatory activity, stress responsiveness, sleep regulation, and cognitive functioning, although the direction and causal significance of these relationships remain uncertain. Within the proposed framework, such changes could contribute to subsequent vulnerability and potentially participate in self-reinforcing feedback processes [15, 30]. Potentially reciprocal associations have also been reported between chronic stress and sleep disruption, inflammation and neural excitability, and cognitive or emotional difficulties and psychosocial stressors [32, 39].

Several alternative explanations should also be considered. For example, recurrent episodes may themselves alter sleep, inflammatory activity, cognition, and functional connectivity; medication exposure and illness chronicity may influence many of the observed biological markers; and some abnormalities may reflect transdiagnostic consequences of stress, disability, or recurrent brain dysfunction rather than disease-specific mechanisms [61, 107, 108]. Longitudinal designs with repeated within-person assessments are needed to clarify temporal ordering and causal relevance.

The potential contribution of this framework is primarily integrative. Whereas epileptogenesis and seizure-network models focus on mechanisms of seizure generation and propagation, and neuroprogression or circadian models focus on the evolution of mood instability, complementary schismogenesis emphasizes the possible reciprocal coupling among biological, psychological, behavioral, and environmental processes. It may therefore complement, rather than replace, existing explanatory models by drawing attention to how compensatory responses may become maladaptive under conditions of recurrent perturbation. Whether this adds predictive value beyond established models remains unknown.

This framework may also provide a way of organizing hypotheses about how different types of interventions produce clinically meaningful benefits. Pharmacological treatments, neuromodulation, psychotherapy, stress-reduction strategies, sleep stabilization, physical activity, and other lifestyle interventions may influence different biological, psychological, behavioral, or environmental processes. Although their immediate mechanisms differ, their clinical effects could be examined in relation to changes in the coordination and flexibility of interconnected biological, psychological, and behavioral processes [40].

Importantly, this interpretation does not imply that BD and epilepsy share identical pathophysiological mechanisms. Rather, it suggests that potentially comparable principles of network instability, compensation, and reciprocal reinforcement may operate in different neural systems and clinical contexts. The specific circuits involved, the biological mechanisms affected, and the clinical manifestations are likely to differ between the two disorders.

At present, the schismogenesis hypothesis should be regarded as a conceptual framework rather than an established explanatory model. Direct empirical evidence specifically supporting this formulation remains limited, and several of its predictions require formal testing [40].

A more empirical evaluation of this framework requires translating its central concepts into measurable variables. Candidate measures that could be used to operationalize reduced regulatory capacity include altered excitation–inhibition dynamics assessed through EEG-derived measures, cortical excitability paradigms, magnetic resonance spectroscopy, or molecular markers related to GABAergic and glutamatergic function [109]. At the network level, the model can be examined using repeated measures of functional connectivity, network flexibility, modularity, synchronization, and temporal variability in connectivity states [110, 111]. At the systemic level, potentially relevant indicators include inflammatory markers, hypothalamic–pituitary–adrenal axis activity, sleep and circadian metrics, autonomic measures, cognitive performance, stress exposure, and digitally derived behavioral variability [58, 112].

The framework also generates specific longitudinal predictions. First, if the framework is valid, increasing instability in neural, physiological, or behavioral regulation should precede at least some seizures or mood episodes rather than merely accompany them [113]. Second, stronger or increasingly maladaptive coupling among sleep disruption, stress-related activity, inflammatory signaling, and network dysregulation should be associated with a higher probability of clinical transition [114, 115]. Third, recurrent episodes should be followed by persistent or cumulative changes in these measures in at least a subgroup of patients, consistent with progressive vulnerability [116, 117]. Finally, effective interventions would be expected to be associated with changes consistent with greater regulatory flexibility, such as improved biological rhythm stability, reduced maladaptive cross-system coupling, or more adaptive network dynamics [58]. These predictions should be examined prospectively using multimodal repeated measures designs rather than inferred from cross-sectional associations [111].

A formal computational formulation may help evaluate whether the proposed reciprocal processes have explanatory or predictive value. One possible approach is to represent each individual as occupying a dynamic state space defined by interacting neural, biological, behavioral, and environmental variables. Within this framework, relative clinical stability could be represented as a resilient attractor state, whereas seizures and mood episodes could be modeled as distinct forms of transition toward disorder-specific pathological states after cumulative perturbations exceed individual thresholds [11]. Relevant state variables may include excitation–inhibition dynamics, temporal variability in functional connectivity, inflammatory and neuroendocrine activity, sleep–circadian regularity, cognitive performance, stress exposure, and digitally measured behavioral change [47, 118].

Dynamical-systems models could be explored as methods for estimating resilience, candidate early-warning signals, critical slowing down, and changes in the stability of symptom-related states over time [119]. Hidden-state models may also help identify latent periods of increased seizure or mood-episode risk that are not fully captured by cross-sectional symptom ratings [102, 119, 120]. Bayesian hierarchical models could further integrate repeated multimodal observations while accounting for between-person heterogeneity, missing data, and uncertainty in individual predictions. Such approaches may be particularly useful because the relative contribution of biological, psychological, and environmental variables is likely to differ substantially across patients and across illness phases.

The framework generates a computationally testable hypothesis: in at least a subgroup of patients, increasing instability within or between these domains should precede clinical transitions, whereas successful treatment should be associated with greater stability, reduced maladaptive coupling, or increased resilience of the non-episode state.

Future longitudinal studies combining neuroimaging, electrophysiology, cognitive assessment, biological markers, and digital monitoring may help determine whether the proposed reciprocal amplification processes have explanatory or predictive relevance for recurrence, illness course, and treatment response in BD and epilepsy [104, 121].

An important limitation of the interpretations presented in this section is that much of the available evidence remains indirect. Similar abnormalities observed in BD and epilepsy do not necessarily imply identical causal mechanisms [122]. For example, alterations in large-scale connectivity, inflammatory signaling, sleep regulation, or cognitive functioning may reflect potentially overlapping mechanisms, disorder-specific consequences, medication effects, illness chronicity, or nonspecific markers of recurrent brain dysfunction [123, 124, 125]. Therefore, the proposed systems-level perspective should be regarded as a heuristic framework that integrates direct, indirect, and theoretical evidence while recognizing that the causal links among these levels remain to be established.

## Clinical Implications and Future Directions

An important implication concerns patient assessment and monitoring. Traditional approaches often rely on cross-sectional evaluations of current symptoms or diagnostic categories. However, measures related to network function, neuroplasticity, inflammation, stress regulation, and sleep may vary over time and across illness phases. Longitudinal monitoring may therefore provide information not captured by isolated assessments, although its incremental clinical value remains to be established [13, 47, 121].

Combining clinical evaluation with repeated measures of cognition, emotional regulation, sleep, stress exposure, and functioning may help characterize within-person changes associated with increasing vulnerability. Whether these changes can reliably anticipate seizures or mood episodes and support earlier intervention requires prospective validation.

Biological and digitally derived markers represent another important area of development. Advances in neuroimaging, electrophysiology, digital phenotyping, and wearable technologies offer complementary opportunities to study illness trajectories across laboratory, clinical, and real-world settings [32]. In epilepsy, repeated or prolonged monitoring of neural activity contributes to diagnosis and treatment planning in selected clinical contexts. In BD, research increasingly focuses on candidate indicators of mood instability, circadian disruption, behavioral variability, and cognitive change [121]. Future studies should determine whether combining these measures improves prediction of relapse, treatment response, or long-term outcomes beyond standard clinical assessment (see Table 3).

**Table 3.** **Candidate translational markers and testable predictions in bipolar disorder and epilepsy**.

Domain	Candidate indicators	Temporal prediction	Hypothesized change with effective intervention
Excitation–inhibition regulation	EEG measures, epileptiform activity, cortical excitability, GABA/glutamate markers	Reduced inhibitory control or increasing instability may precede clinical transitions	Reduced pathological excitability and improved regulatory balance
Network dynamics	Functional connectivity, synchronization, network flexibility, modularity	Increasing connectivity instability or maladaptive coupling may signal vulnerability	Greater network stability and adaptive flexibility
Inflammation and stress regulation	CRP, cytokines, cortisol, heart-rate variability	Stronger inflammatory or stress-related activation may accompany high-risk periods	Reduced inflammatory and stress-related dysregulation
Sleep and circadian rhythms	Sleep duration and variability, actigraphy, social-rhythm regularity	Sleep loss, rhythm irregularity, or circadian disruption may precede seizures or mood episodes	More regular sleep–wake patterns and improved rhythm stability
Cognitive and emotional regulation	Executive function, affective lability, emotion-regulation measures	Deterioration in cognitive control or emotional regulation may indicate reduced resilience	Improved cognitive efficiency and emotional regulation
Behavioral and digital indicators	Activity, mobility, communication patterns, ecological momentary assessment	Increasing behavioral variability or social withdrawal may identify latent high-risk states	Stabilization of daily routines and reduced behavioral variability
Clinical indicators	Seizure diaries, mood ratings, prodromal symptoms, adherence, stress exposure	Accumulation of warning signs may precede overt episodes	Reduced episode frequency, severity, and duration

Note: These variables should be considered candidate translational indicators rather than validated biomarkers. Their relevance is likely to vary according to epilepsy subtype, bipolar illness phase, treatment exposure, and individual clinical characteristics. Prospective multimodal studies are required to determine their predictive validity and feasibility in routine care. GABA, Gamma-Aminobutyric; CRP, C-reactive protein; EEG, Electroencephalographic.

The reviewed evidence highlights the potential value of more personalized assessment and treatment. Patients with similar diagnoses may differ substantially in genetic susceptibility, cognition, inflammatory burden, stress sensitivity, lifestyle, and network organization. Such heterogeneity may contribute to, or be associated with, differences in illness course and therapeutic response, and identifying reproducible and clinically meaningful subgroups could eventually support more targeted interventions. Personalization should not, however, involve extrapolating treatments from epilepsy to BD, or vice versa, solely because the disorders may share some mechanisms. Clinical decisions must remain grounded in condition-specific evidence and guidelines, while considering whether modifiable transdiagnostic factors such as sleep disruption, stress, cognitive difficulties, or lifestyle instability require additional intervention.

Prevention is another relevant implication. Chronic stress, sleep disruption, sedentary behavior, social isolation, and substance misuse are potentially modifiable factors associated with less favorable illness course or recurrence in one or both disorders. Although unlikely to be primary causes, they may influence illness expression through interactions with biological vulnerability. Interventions targeting sleep, stress, lifestyle, and social support may complement pharmacological treatment and improve selected clinical or functional outcomes, although their effects on long-term recurrence remain uncertain [69, 85].

Several research questions remain unresolved. The causal relationships among network dysfunction, neuroplasticity, inflammation, and clinical symptoms are still unclear. The mechanisms underlying heterogeneity within both disorders also require further study. In addition, longitudinal research is needed to determine whether longitudinal biological and network changes precede clinical deterioration or mainly reflect its consequences.

The potential translational value of the framework lies not in proposing uniformly shared biomarkers or interventions, but in identifying domains in which repeated multimodal assessment may help identify candidate indicators of increasing vulnerability, characterize individual trajectories, and examine whether treatment is associated with restoration of regulatory stability [121]. This may complement disorder-specific models by focusing on dynamic processes that may precede, accompany, or follow overt seizures or mood episodes [47].

A major limitation is the marked heterogeneity of both conditions. Potentially comparable dimensions may be particularly relevant to subgroups with recurrent or treatment-resistant illness, prominent sleep or circadian disruption, cognitive impairment, high stress sensitivity, or specific epilepsy-network phenotypes [43, 100]. Future studies should therefore avoid treating BD and epilepsy as unitary categories and examine whether biomarkers, network signatures, and treatment-response patterns vary by bipolar subtype, mood polarity, epilepsy etiology, seizure type, epileptogenic localization, illness stage, and medication exposure.

Future research should also complement studies of individual mechanisms with designs integrating genetic, neuroimaging, neurophysiological, cognitive, behavioral, and environmental data. Computational neuroscience, dynamical-systems modelling, Bayesian hierarchical analysis, machine learning, and digital health may help model candidate latent risk states, quantify within-person changes in resilience, and integrate multimodal predictors of clinical transition. However, these approaches require prospective validation, transparent reporting, external replication, assessment of calibration and clinical utility, and demonstration of incremental value before routine clinical use [98, 121].

## Conclusions

BD and epilepsy are clinically distinct and highly heterogeneous conditions, but available evidence suggests that some patient subgroups may show comparable or partially overlapping clinical and biological features related to recurrent episodes, altered in network function or regulation, sleep and circadian disruption, stress sensitivity, cognitive impairment, neuroplasticity, and treatment response. The exploratory systems-level framework proposed in this review is intended to organize these observations and generate testable hypotheses regarding vulnerability, recurrence, and treatment response. Future longitudinal, multimodal, and subtype-stratified studies are needed to determine whether combined clinical, neurophysiological, biological, behavioral, and digital measures can improve individualized risk estimation, monitoring, and clinical decision-making beyond standard assessment.

## Availability of Data and Materials

Not applicable.
